# Supplementary material for: Engineering multiple species-like genetic incompatibilities in insects
Source: Nat Commun. 2020 Sep 8;11:4468. doi: 10.1038/s41467-020-18348-1 (PMC7478965; doi:10.1038/s41467-020-18348-1)
Supplement: Supplementary file 1 — Supplementary Information [file 41467_2020_18348_MOESM1_ESM.pdf]

Supplementary Information for:

Engineering multiple species-like genetic incompatibilities in insects

Maselko et al.

This PDF file includes:

Supplementary Figures 1 to 10
Supplementary Tables 1 to 4
References

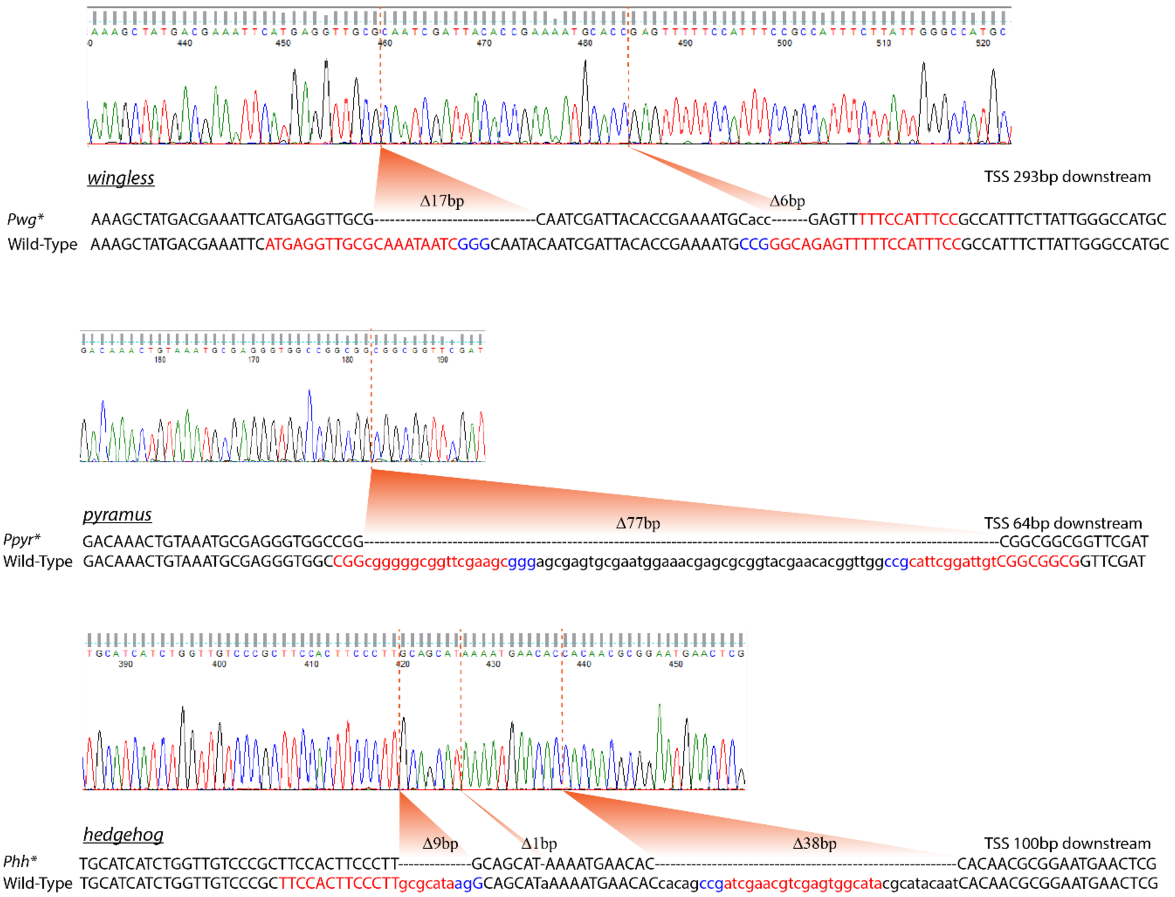

Supplementary Figure 1. Promoter mutation characterization. Promoter mutant sequencing traces and alignments to wild-type promoters. Targeted protospacers indicated in red and PAMs indicated in blue.

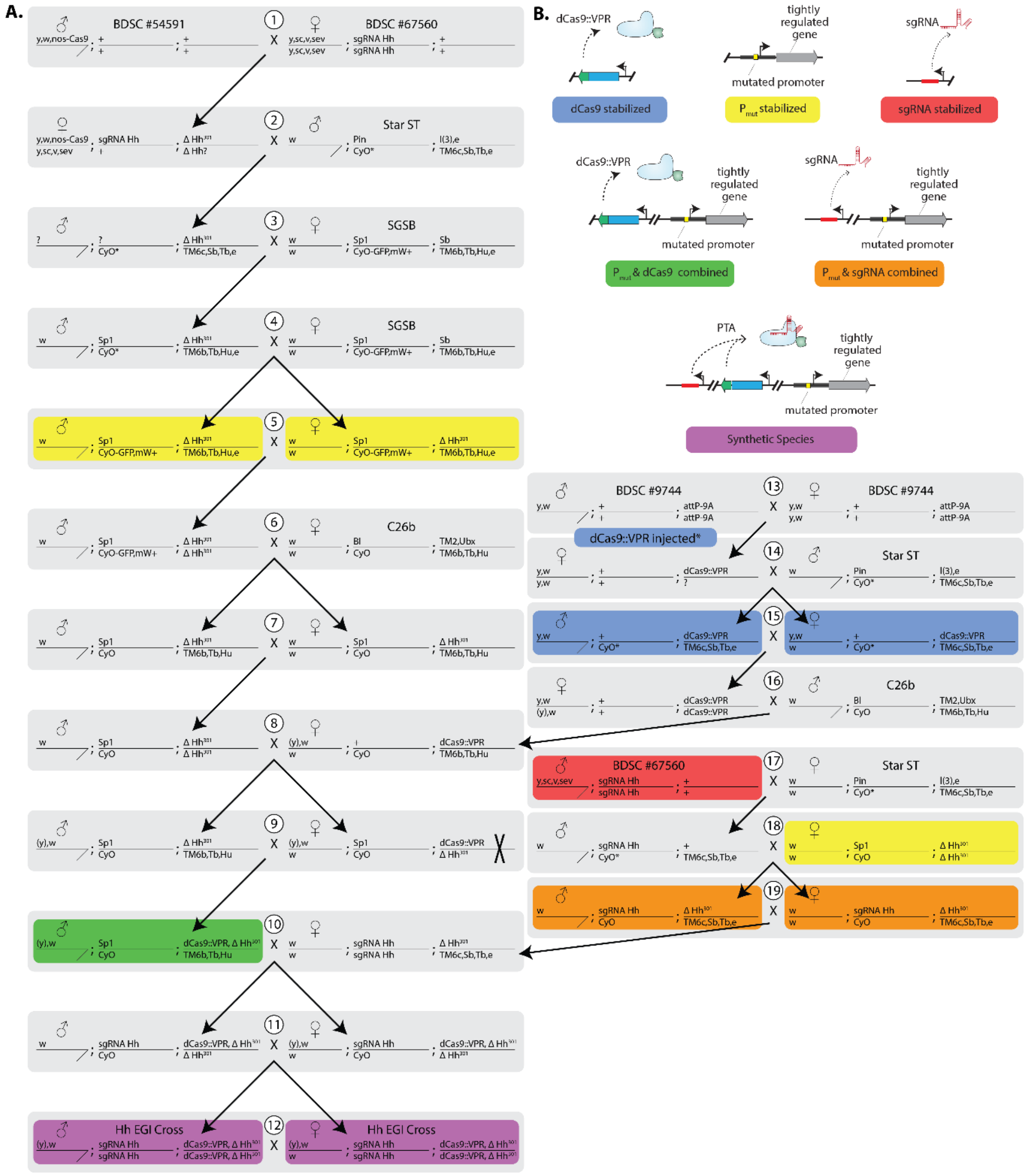

Supplementary Figure 2. Crossing strategy to produce *hh*-EGI flies. (A.) Genotypes and sex of flies involved in crosses required to bring together EGI components. Crosses are indexed with numbered white circles. ‘X’ designates a recombination event required in the female parent of cross #9. The female from cross #18 resulted from cross #7 (not cross #5). Embryos from cross #13 were injected with promoter::dCas9::VPR constructs and ΦC31 integrase. Question mark denotes a chromosome genotype that was not verified. (B.) Illustrated and color-coded genotypes of key intermediates. BDSC #54591, BDSC #67560, and BDSC #9744 were purchased from the Bloomington Drosophila Stock Center. Star ST, SGSB, and C26b are balancer strains.

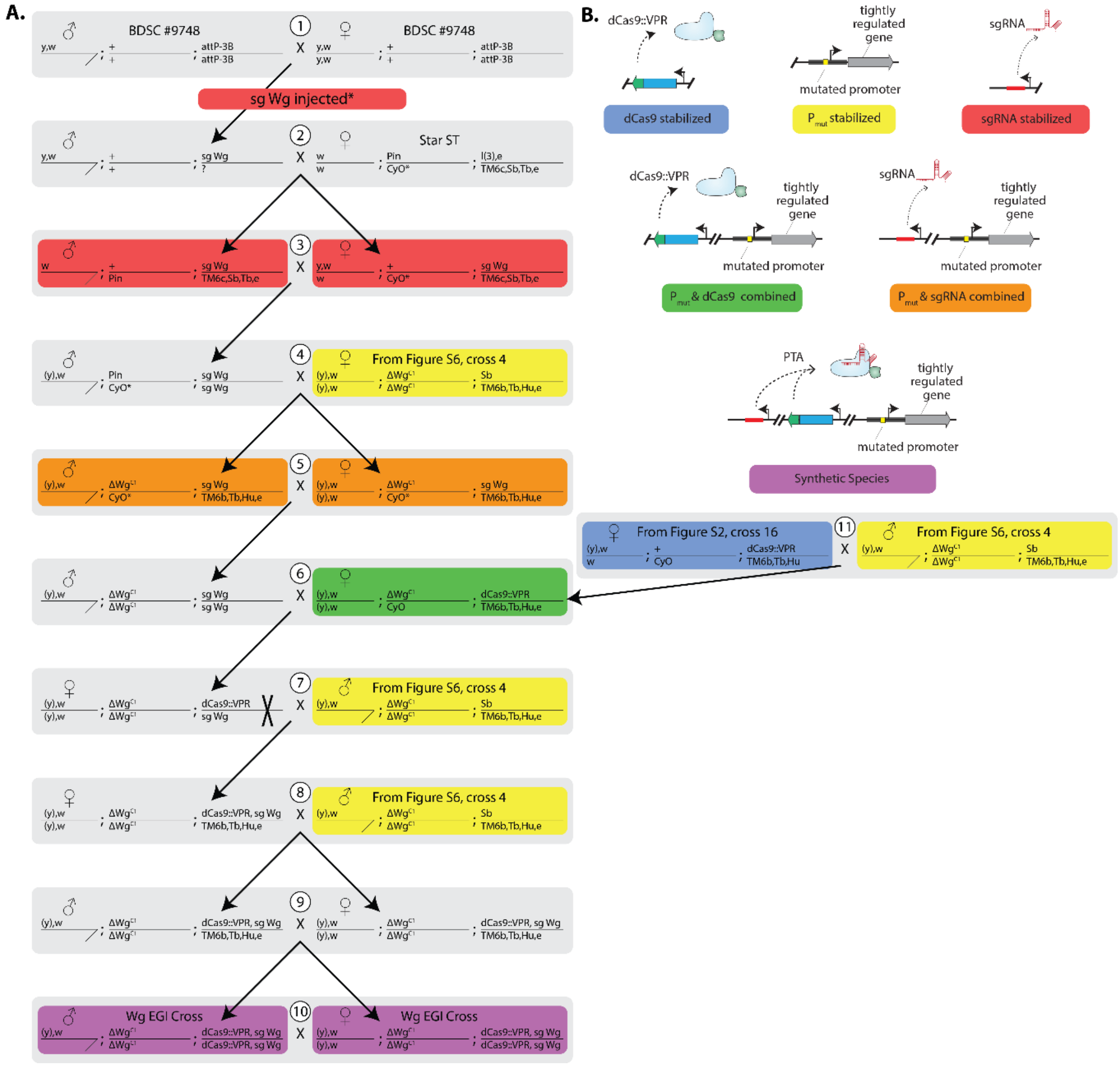

Supplementary Figure 3. Crossing strategy to produce *wg*-EGI flies. (A.) Genotypes and sex of flies involved in crosses required to bring together EGI components. Crosses are indexed with numbered white circles. 'X' designates a recombination event required in the female parent of cross #7. Embryos from cross #1 were injected with a sgRNA-*wg* construct and ΦC31 integrase. Question mark denotes a chromosome genotype that was not verified. The males in crosses #7, 8, and 11 and the female in cross #4 are offspring from Supplementary Figure 6, cross #4. The female in cross #11 is offspring from Supplementary Figure 2, cross #16. (B.) Illustrated and color-coded genotypes of key intermediates. BDSC #9748 was purchased from the Bloomington Drosophila Stock Center. Star ST is a balancer strain.

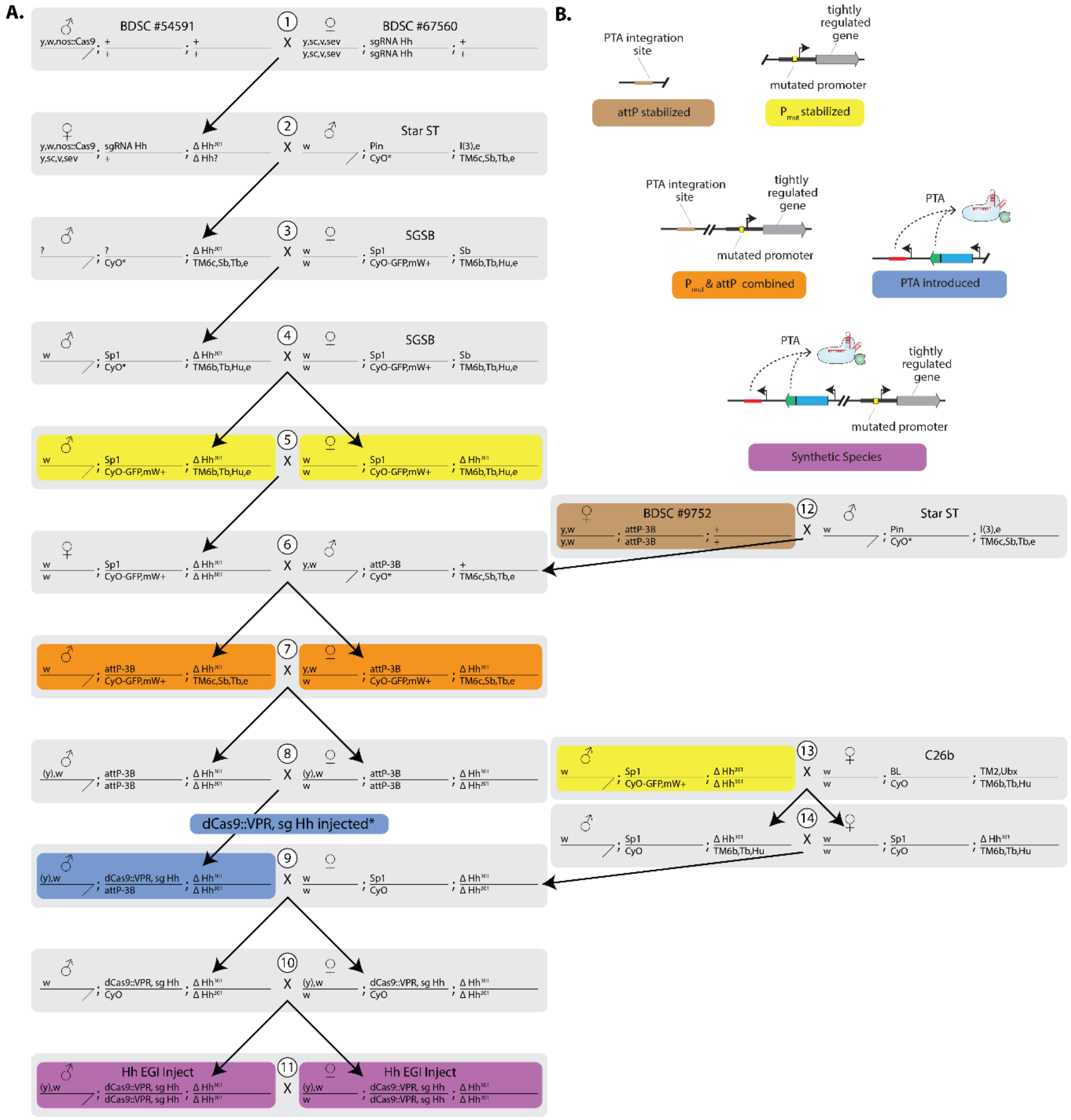

Supplementary Figure 4. Reinjection strategy to produce *hh*-EGI flies. (A.) Genotypes and sex of flies involved in crosses required to bring together EGI components. Crosses are indexed with numbered white circles. Embryos from cross #8 were injected with promoter::dCas9::VPR + sgRNA-*hh* constructs and ΦC31 integrase. Question mark denotes a chromosome genotype that was not verified. (B.) Illustrated and color-coded genotypes of key intermediates. BDSC #54591, BDSC #67560, and BDSC #9752 were purchased from the Bloomington Drosophila Stock Center. Star ST, SGSB, and C26b are balancer strains.

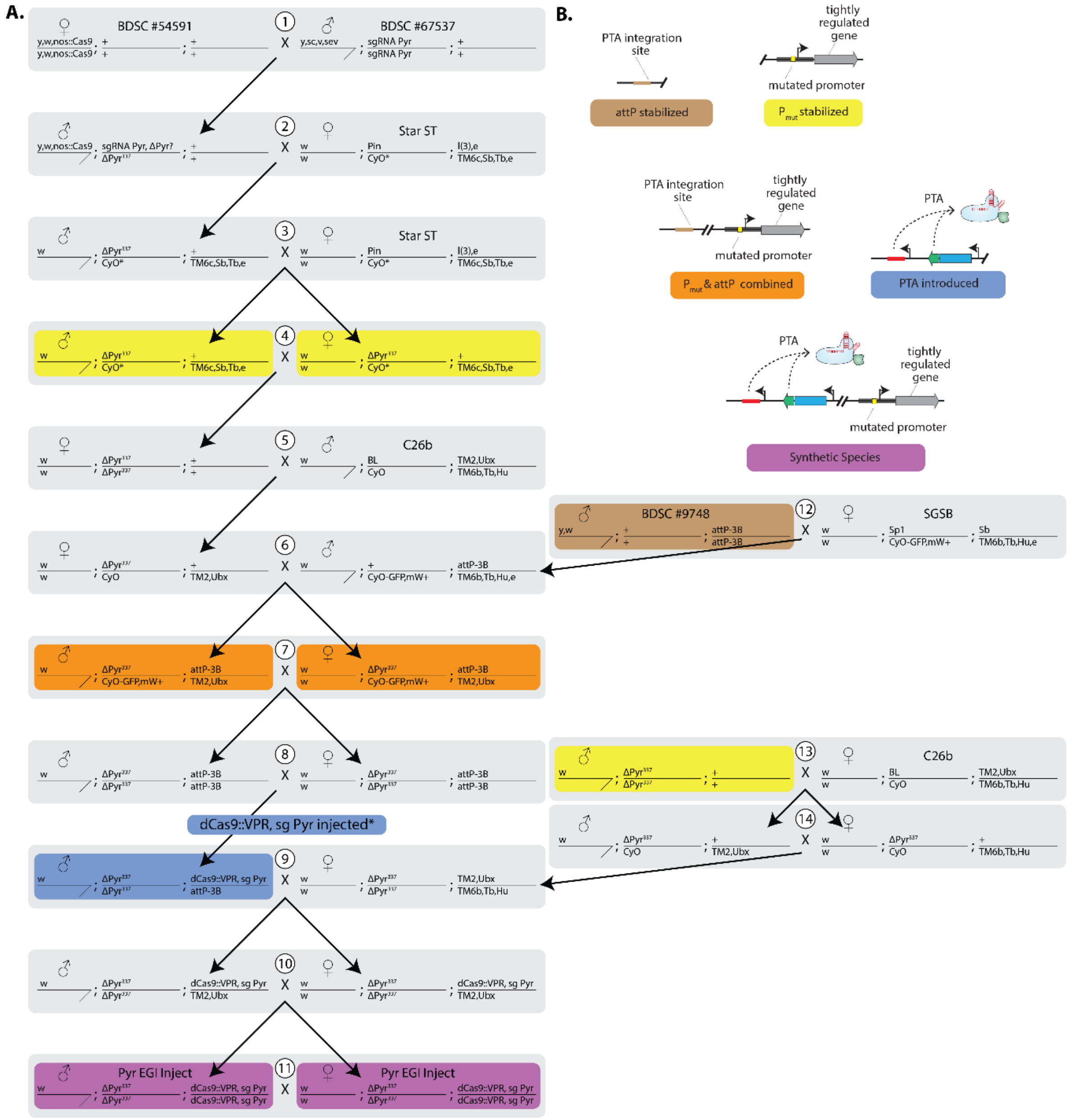

Supplementary Figure 5. Reinjection strategy to produce *pyr*-EGI flies. (A.) Genotypes and sex of flies involved in crosses required to bring together EGI components. Crosses are indexed with numbered white circles. Embryos from cross #8 were injected with promoter::dCas9::VPR + sgRNA-*pyr* constructs and ΦC31 integrase. Question mark denotes a chromosome genotype that was not verified. (B.) Illustrated and color-coded genotypes of key intermediates. BDSC #54591, BDSC #67537, and BDSC #9748 were purchased from the Bloomington Drosophila Stock Center. Star ST, SGSB, and C26b are balancer strains.

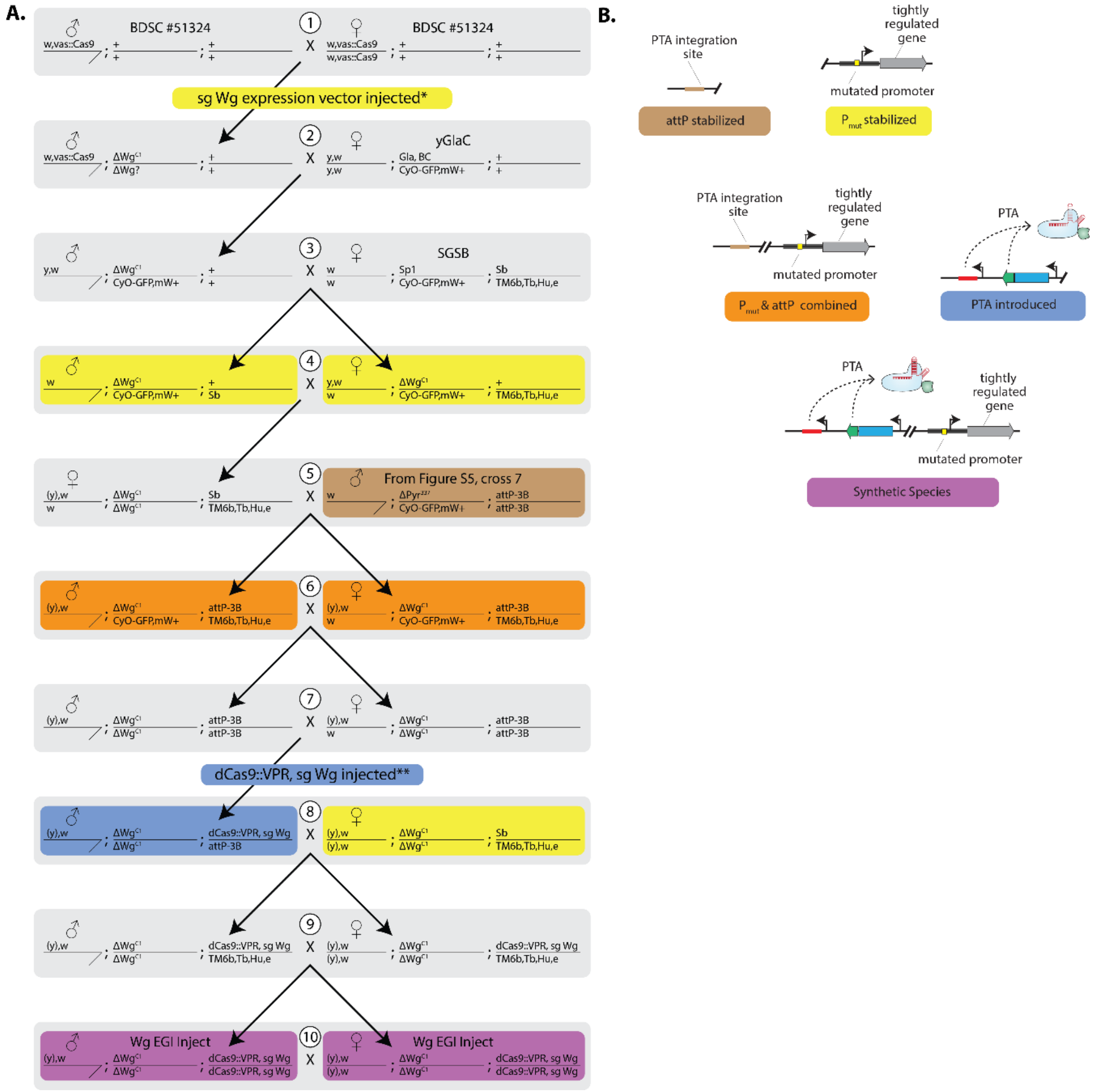

Supplementary Figure 6. Reinjection strategy to produce *wg*-EGI flies. (A.) Genotypes and sex of flies involved in crosses required to bring together EGI components. Crosses are indexed with numbered white circles. Embryos from cross #1 were injected with the sgRNA-*wg* expression construct. Embryos from cross #7 were injected with promoter::dCas9::VPR + sgRNA-*wg* constructs and ΦC31 integrase. Question mark denotes a chromosome genotype that was not verified. The male in cross #5 is offspring from Supplementary Figure 5, cross #7. (B.) Illustrated and color-coded genotypes of key intermediates. BDSC #51324 was purchased from the Bloomington Drosophila Stock Center. yGlac and SGSB are balancer strains.

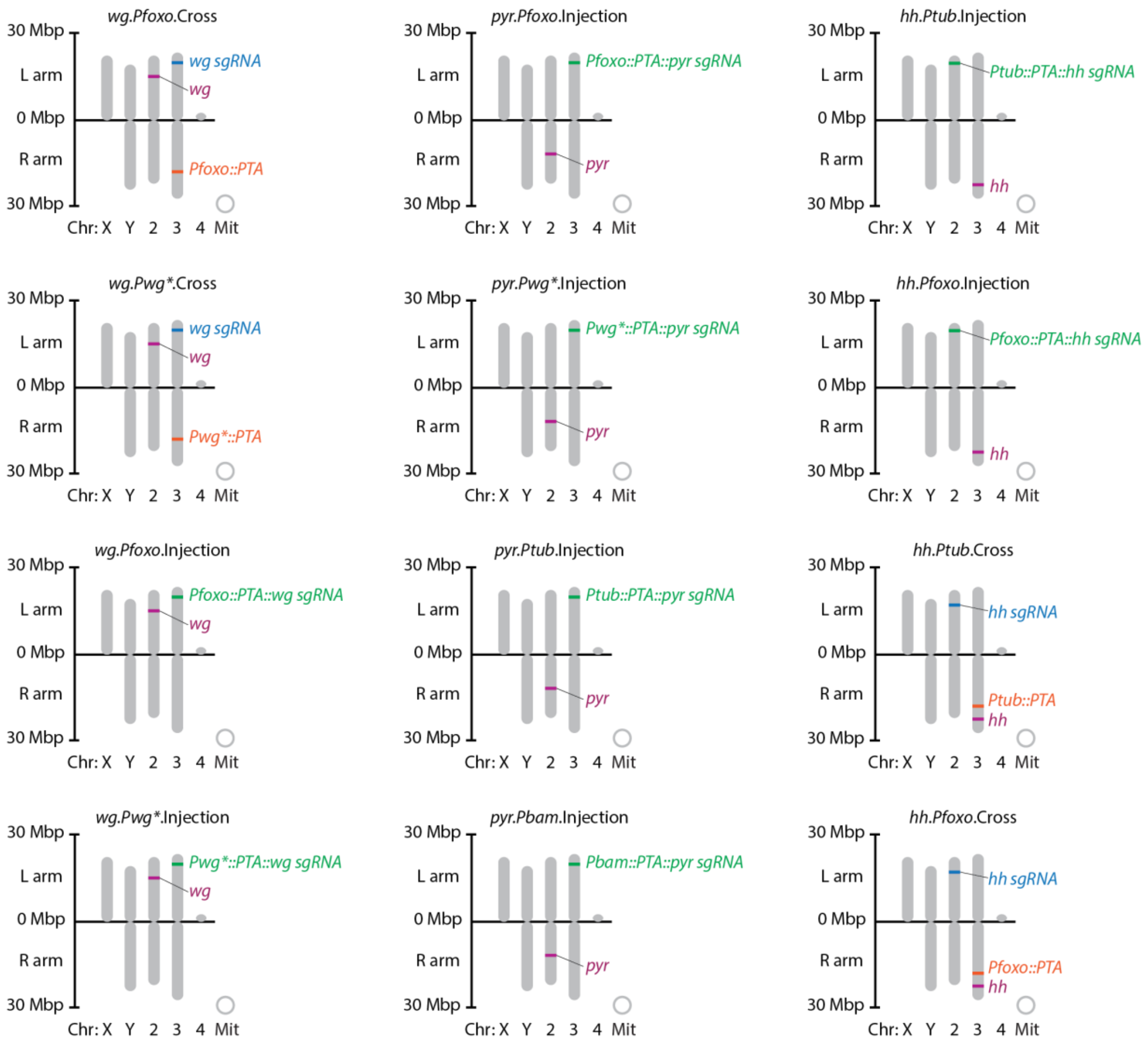

Supplementary Figure 7: Chromosomal maps of all EGI strains reported in this work.

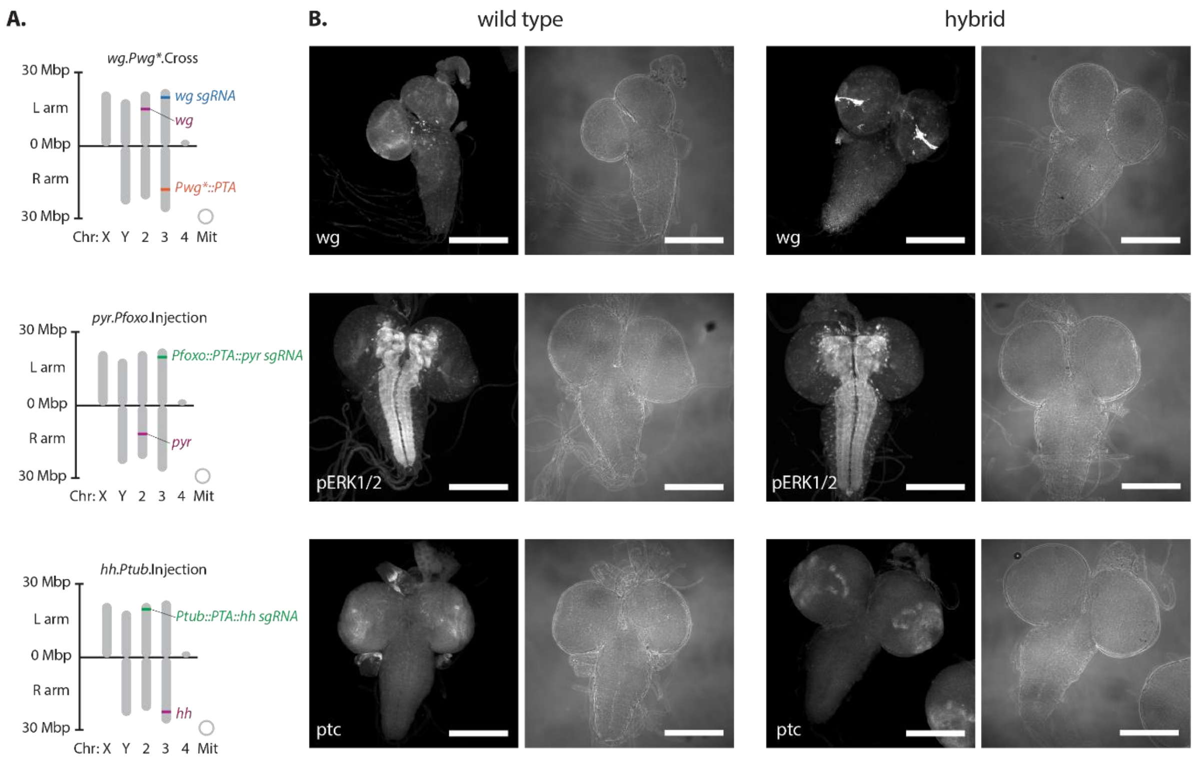

Supplementary Figure 8: Immunohistochemistry of dissected brains. (A.) Chromosomal locations of genome alterations for EGI strains whose hybrid offspring were analysed by immunohistochemistry. EGI strains illustrated here correspond to those used in Figure 3. (B.) Immunofluorescence staining of 3rd instar larval brains from wild-type (left) or hybrid (right) showing over- or ectopic-expression of targeted signalling pathways. Grayscale images show antibody staining for proteins encoded by lethal overexpression target (*wingless*, top) or downstream signalling pathway components (p-*ERK1/2*, middle and *patched*, bottom). Corresponding brightfield images of the brains to the right. Scale bar = 200 µm. Images are representative of at least six independent biological replicates for each strain.

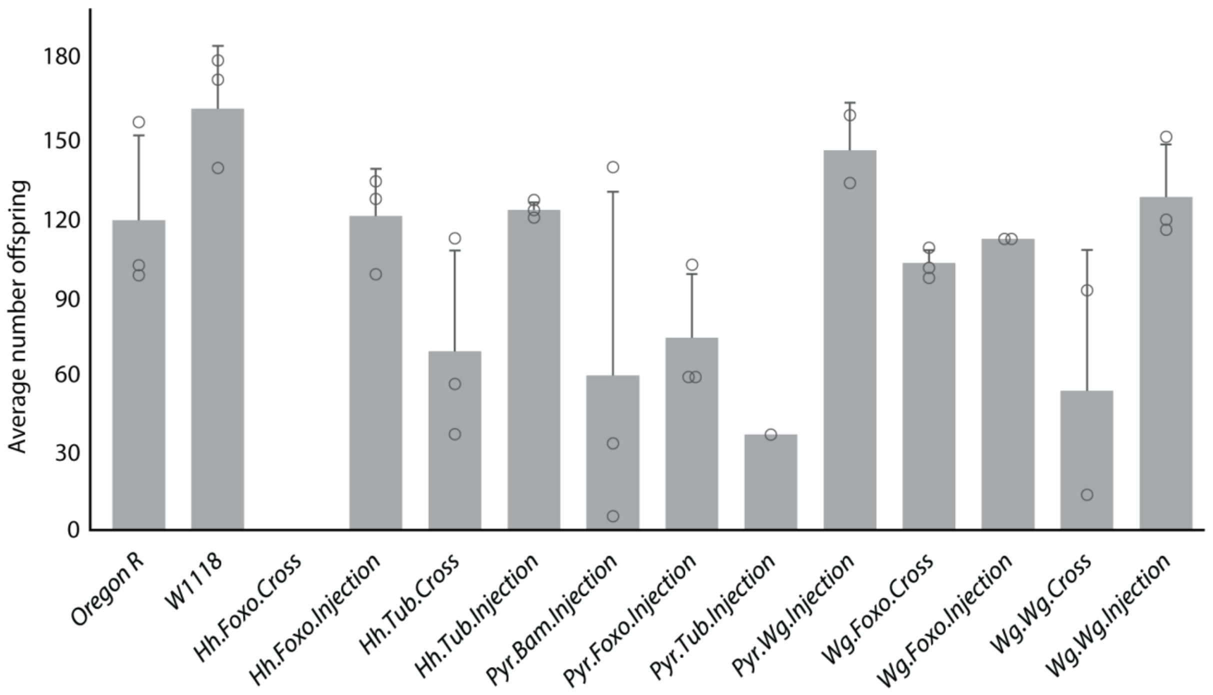

Supplementary Figure 9: Fecundity of EGI and WT strains when self-mated. Average adult offspring resulting from three matings between two males and three females of the same strain. Strains are in the same order and contain the same data as in the mating compatibility assay (Figure 4). Error bars denote one standard deviation across three replicates, unless otherwise stated. Hh.Foxo.Cross had no replicates. Pyr.Tub.Injection contained only 1 replicate. Pyr.Wg.Injection, Wg.Foxo.Injection, and Wg.Wg.Cross contained 2 replicates each.

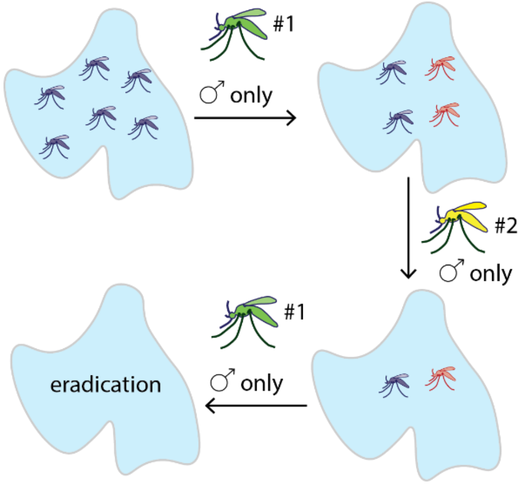

Supplementary Figure 10: Iterative release scheme for negatively correlating cross resistance. Purple denotes wild-type pests, green and yellow denote mutually-incompatible EGI strains, for which only males would be released. Orange denotes resistant 'escapees', which inherit half of their genome from the previously released biocontrol EGI strain.

Supplementary Table 1: Promoter Target Sequences

Target Promoter	Target 1	Target 2	Reference
hh	TATGCCACTCGACGTTCGAT CGG	TTCCACTTCCCTTGCGCATA AGG	[1]
hid	CACATGCACGTGCATGA AGG	CATGCACGTGCATGAAGG AGG	[2]
pyr	CGGCGGGGGCGGTTCGAAGC GGG	CGCCGCCGACAATCCGAATG CGG	[1]
upd1	GCGAGCTGAGCGGCCTCTGC CGG	TGCCGACGAGCGGTACGCCA TGG	[1]
upd2	CAGCAAGCGATTGTGATAGT TGG	TGCTGATGCTGATCGCTCCA CGG	[1]
upd3	TGGAGTGGAGTGTTGTGGAG TGG	GGAGTTCAGTCAGTCTCCGC CGG	[1]
vn	AGCATTCACAACGTATCTCA CGG	GTGCCGAAGATATTCGAACA CGG	[1]
wg	ATGAGGTTGCGCAAATAATC GGG	GGAAATGGAAAAACTCTGCC CGG	[3]

Supplementary Table 2: *D. melanogaster* strains used in this study

Strain	Description	Reference	BDSC #
Wild Type	Oregon R	[4]	N/A
White Eye	w^{1118}	[5]	5905
3rd Chr. attP9	y^{1} w^{1118}; ; PBac{y[+]-attP-9A}VK00027	[6]	9744
3rd Chr. attP3	y^{1} w^{1118}; PBac{y[+]-attP-3B}VK00031	[6]	9748
2nd Chr. attP3	y^{1} w^{1118}; PBac{y[+]-attP-3B}VK00037	[6]	9752
Vas-Cas9	w[1118]; PBac{y[+mDint2]=vas-Cas9}VK00027	[7]	51324
2nd Chr. attP3, ΔHH^{301}	w^{1118} ;PBac{y[+]-attP-3B}VK00037; ΔHH^{301}	this study	
3rd Chr. attP3, Δpyr^{337}	w^{1118} ;Δpyr^{337}; PBac{y[+]-attP-3B}VK00031	this study	
3rd Chr. attP3, ΔWg^{C1}	w^{1118} ;ΔWg^{C1}; PBac{y[+]-attP-3B}VK00031	this study	
2nd Chr. bal, ΔHH^{301}	w^{1118} ; Sp1/ CyO; ΔHH^{301}	this study	
3rd Chr. bal, Δpyr^{337}	w^{1118} ;Δpyr^{337}; TM2/ TM6b, Tb, Hu	this study	
3rd Chr. bal, ΔWg^{C1}	w^{1118} ;ΔWg^{C1}; Sb/ TM6b, Tb, Hu	this study	
hh.FoxO.cross	w^{1118}; P{y[+t7.7] v[+t1.8]=TOE.GS00191}attP40; P{pFoxo::dCas9-VPR}attP-9A, ΔHH^{301}	this study	
hh.FoxO.injection	y^{1} w^{1118} ; P{pFoxo::dCas9-VPR::sgRNA^{HH}}attP-3B; ΔHH^{301}	this study	
hh.Tub.cross	w^{1118}; P{y[+t7.7] v[+t1.8]=TOE.GS00191}attP40; P{ pTub::dCas9-VPR }attP-9A, ΔHH^{301}	this study	
hh.Tub.injection	y^{1} w^{1118} ; P{pTub::dCas9-VPR::sgRNA^{HH}}attP-3B; ΔHH^{301}	this study	
pyr.Bam.injection	w^{1118} ;Δpyr^{337}; P{pBam::dCas9-VPR::sgRNA^{Pyr}}attP-3B	this study	
pyr.FoxO.injection	w^{1118} ;Δpyr^{337}; P{pFoxo::dCas9-VPR::sgRNA^{Pyr}}attP-3B	this study	
pyr.Tub.injection	w^{1118} ;Δpyr^{337}; P{pTub::dCas9-VPR::sgRNA^{Pyr}}attP-3B	this study	
pyr.Wg.injection	w^{1118} ;Δpyr^{337}; P{pΔWg^{C1}::dCas9-VPR::sgRNA^{Pyr}}attP-3B	this study	
wg.FoxO.cross	w^{1118}; P{sgRNA^{Wg}}attP-3B, ΔWg^{C1}; P{pFoxo::dCas9-VPR}attP-9A	this study	
wg.FoxO.injection	y^{1} w^{1118} ;ΔWg^{C1}; P{pFoxo::dCas9-VPR::sgRNA^{Wg}}attP-3B	this study	
wg.Wg.cross	w^{1118}; PBac{sgRNA^{Wg}}attP-3B, ΔWg^{C1}; P{pΔWg^{C1}::dCas9-VPR}attP-9A	this study	
wg.Wg.injection	y^{1} w^{1118} ;ΔWg^{C1}; P{pΔWg^{C1}::dCas9-VPR::sgRNA^{Wg}}attP-3B	this study	
nos-Cas9	y^{1} M{w[+mC]=nos-Cas9.P}ZH-2A, w*	[8]	54591
hh sgRNA	y^{1} sc* v^{1}; P{y[+t7.7] v[+t1.8]=TOE.GS00191}attP40	[1]	67560
hid sgRNA	y^{1} w^{1118}; PBac{sgRNA^{Hid}}attP-3B	this study	
pyr sgRNA	y^{1} sc* v^{1}; P{y[+t7.7] v[+t1.8]=TOE.GS00085}attP40	[1]	67537
upd1 sgRNA	y^{1} sc* v^{1}; P{y[+t7.7] v[+t1.8]=TOE.GS00169}attP40	[1]	67555
upd2 sgRNA	y^{1} sc* v^{1}; P{y[+t7.7] v[+t1.8]=TOE.GS00171}attP40	[1]	67556
upd3 sgRNA	y^{1} sc* v^{1}; P{y[+t7.7] v[+t1.8]=TOE.GS00129}attP40	[1]	67546
vn sgRNA	y^{1} sc* v^{1}; P{y[+t7.7] v[+t1.8]=TOE.GS00144}attP40	[1]	67548
wg sgRNA	y^{1} w^{1118}; ; PBac{sgRNA^{Wg}}attP-3B	this study	
pTub::dCas9-VPR	y^{1} w^{1118}; ;P{pTub::dCas9-VPR}attP-9A	this study	
pFoxo::dCas9-VPR	y^{1} w^{1118}; ;P{pFoxo::dCas9-VPR}attP-9A	this study	
pTub::dxCas9-VPR	y^{1} w^{1118}; ;P{pTub::dxCas9-VPR}attP-9A	this study	
pΔWg::dCas9-VPR	y^{1} w^{1118}; ;P{pΔWg^{C1}::dCas9-VPR}attP-9A	this study	
ΔWg	w^{1118}; ΔWg^{C1}	this study	
ΔHH	w^{1118}; ; ΔHH^{301}	this study	
ΔPyr	w^{1118}; ΔPyr^{337}	this study	

Supplementary Table 3: Plasmids used in this study

Plasmid	Integrated in Fly Strain	Description	Reference/ GenBank Accession #
pMM7-1-1	N/A	pU6-BbsI-chiRNA targeting upstream wg promoter protospacer	this study
pMM7-1-2	N/A	pU6-BbsI-chiRNA targeting downstream wg promoter protospacer	this study
pMM7-5-3	wg sgRNA<br>wg.FoxO.cross	Expresses dual sgRNAs targeting Wg promoter	this study
pMM7-6-1	N/A	dCas9-VPR plasmid. Promoters cloned into NotI restriction site.	this study/ MT882253
pMM7-6-2	hh.FoxO.cross<br>wg.FoxO.cross<br>wg.Wg.cross	Foxo promoter driving dCas9-VPR expression	this study
pMM7-6-3	pTub::dCas9-VPR<br>hh.Tub.cross	Short tubulin alpha promoter driving dCas9-VPR expression	this study
pMM7-6-4	$p\Delta Wg^{C1}$::dCas9-VPR<br>wg.Wg.cross	Mutated Wg promoter driving dCas9-VPR expression	this study
pMM7-9-1	N/A	dxCas9-VPR plasmid. Promoters cloned into NotI restriction site.	this study/ MT882254
pMM7-9-4	pTub::dxCas9-VPR	Short tubulin alpha promoter driving d**X**Cas9-VPR expression	this study
pMM7-6-2-HH	hh.FoxO.injection	Foxo promoter driving dCas9-VPR expression. Dual sgRNAs targeting HH promoter.	this study
pMM7-6-2-Pyr	pyr.FoxO.injection	Foxo promoter driving dCas9-VPR expression. Dual sgRNAs targeting Pyr promoter.	this study
pMM7-6-2-Wg	wg.FoxO.injection	Foxo promoter driving dCas9-VPR expression. Dual sgRNAs targeting Wg promoter.	this study
pMM7-6-3-HH	hh.Tub.injection	Short tubulin alpha promoter driving dCas9-VPR expression. Dual sgRNAs targeting HH promoter.	this study
pMM7-6-3-Pyr	pyr.Tub.injection	Short tubulin alpha promoter driving dCas9-VPR expression. Dual sgRNAs targeting Pyr promoter.	this study
pMM7-6-4-Pyr	pyr.Wg.injection	Mutated Wg promoter driving dCas9-VPR expression. Dual sgRNAs targeting Pyr promoter.	this study
pMM7-6-4-Wg	wg.Wg.injection	Mutated Wg promoter driving dCas9-VPR expression. Dual sgRNAs targeting Wg promoter.	this study
pMM7-6-5-Pyr	pyr.Bam.injection	Bam promoter driving dCas9-VPR expression. Dual sgRNAs targeting Pyr promoter.	this study
pU6-BbsI-chiRNA	N/A	sgRNA expression plasmid	[7]
pAct:dCas9-VPR	N/A	Source of dCas9-VPR	[9]
pCFD4	N/A	sgRNA expression plasmid	[8]
p{CFD4-3xP3::DsRed}	N/A	sgRNA expression plasmid	[10]

Supplementary Table 4: Primer Sequences

Name	Sequence (5’ -> 3’)	Notes
MM_2step_dCas_F	ctggtctagagcccgggcgGGCTAGCCAATTCTATATTCTAAAAACAC	Ampliflifies dCas9-VPR from pAct:dCas9-VPR
MM_2step_dCas_R	ctgattatgatctagagtcgGTCAAAACAGAGATGTGT	
MM_bgl_SVT_F	tctggtctagagcccgggcgGCTAGCcgactctagatcataatcag	Amplifies SV40 terminator from ph-Stinger
MM_bgl_SVT_R	cggggtaccggcgcgccggcgtaagatacattgatgagtttg	
MM_pFoxo_F	ACGTAAGTACTGTCTGCAGCGTAAGCTTCGTACGTAGC gtcaaatttggttgtgattac	Amplifies Foxo promoter and 5'UTR
MM_pFoxo_R	AGCCCAATGGAGTACTTCTTGTCCATtttgtaagattGC tccaattcgctttttattcg	
MM_sptuba_F2	GCAGCGTAAGCTTCGTACGTAGCgaccgtctcaaagtactgccT	Amplifies portion of tubulin promoter and 5' UTR
MM_sptuba_R	GGAGTACTTCTTGTCCATtttgtaagattGCattggaagtgtttcacacgcgac	
MM_pWGdcas_F	ACGTAAGTACTGTCTGCAGCGTAAGCTTCGTACGTAGC cagctccttgttggttgac	Amplifies wingless promoter and 5' UTR
MM_pWGdcas_R	AGCCCAATGGAGTACTTCTTGTCCATtttgtaagattGC gctgatcgggtttatctgttc	
MM_pBam_F	ACGTAAGTACTGTCTGCAGCGTAAGCTTCGTACGTAGC actctaaaacgtaaagaaac	Amplifies bam promoter and 5'UTR
MM_pBam_R	AGCCCAATGGAGTACTTCTTGTCCATtttgtaagattGC cttaagttaaatcacacaaa	
MM_2wg_F	AGATATCCGGGTGAACTTCG ATGAGGTTGCGCAAATAATC GTTTTAGAGCTAGAAATAGC	Primers to clone wingless spacers into p{CFD4-3xP3::DsRed}
MM_2wg_R	GCTATTTCTAGCTCTAAAAC GGCAGAGTTTTTCCATTTCC CGACGTTAAATTGAAAATAG	
MM_dmsgRNA_F	tcttaGCGGCTCGAGGGTACAAGCCGAATTGATCCACTAG	Amplifies sgRNA cassetes.

MM_dmsgRNA_R	TAGTGGATCTCTAGAGGTACATAATAAATACTGGCGAAAGC	
Promoter sequencing		
HH_F	CCAGGAGTCACACAATACAC	Hedghog promoter
HH_R	GCGAATACGAATGCGAGTAT	Hedghog promoter
Pyr_F	GAACGAACTGGCCCACTTGG	Pyramus promoter
Pyr_R	CTGTAGCCGCGCAATGCACT	Pyramus promoter
Wg_F	CGGAATGCCAAAGTGTGT	Wingless promoter
Wg_R	GCTAGTTATAGATCGGTTCGATC	Wingless promoter
Primers and gBlock to generate dXCas9-VPR		
MM_xCas1_F2	ACGTAAGTACTGTCTGCAGCGTAAGCTTCGTACGTAGCGGCCGCaatcttacaaaATGGACAAGAAGTACTCCATTG	
MM_xCas1_R	GTAGGTGTCTTTGCTCAGTTGAAGCTTGGTATCTTCGGCCAGGTCGAA	
MM_xCas2_F	TCCGATGGATTTGCCAACCGGAACTTCATTCAGTTGATCCATGATGACTCTCTC	
MM_xCas2_R	CTCGTTACCTTTCTGCAGCACGCCCGCACTAGCGA	
MM_xCas3_F	CGAATGCTCGCTAGTGCGGGCGTGCTGCAGAAAGGTAACGAG	
MM_xCas3_R	tgattatgatctagagtcggTCAAAACAGAGATGTGTCGAAG	
MM_xCas4_F	TTCGACACATCTCTGTTTTGAccgactctagatcataatc	
MM_xCas4_R	ACACTAGTGGATCTCTAGAGGTACCCTCGAGCCGCtaagatacattgatgagttt	

MM_gdXCas9

ACCAAGCTTCAACTGAGCAAAGACACCTACGATGATGATCTCGACAATCTGCTGGCCCAGATCGGCG
ACCAGTACGCAGACCTTTTTTTGGCGGCAAAGAACCTGTCAGACGCCATTCTGCTGAGTGATATTCTG
CGAGTGAACACGGAGATCACCAAAGCTCCGCTGAGCGCTAGTATGATCAAGCTCTATGATGAGCACC
ACCAAGACTTGACTTTGCTGAAGGCCCTTGTCAGACAGCAACTGCCTGAGAAGTACAAGGAAATTTT
CTTCGATCAGTCTAAAAATGGCTACGCCGGATACATTGACGGCGGAGCAAGCCAGGAGGAATTTTAC
AAATTTATTAAGCCCATCTTGGAAAAAATGGACGGCACCGAGGAGCTGCTGGTAAAGCTTAACAGAG
AAGATCTGTTGCGCAAACAGCGCACTTTCGACAATGGAATCATCCCCCACCAGATTCACCTGGGCGA
ACTGCACGCTATCCTCAGGCGGCAAGAGGATTTCTACCCCTTTTTGAAAGATAACAGGGAAAAGATT
GAGAAAATCCTCACATTTCGGATACCCTACTATGTAGGCCCCCTCGCCCGGGGAAATTCCAGATTCGC
GTGGATGACTCGCAAATCAGAAGAAACCATCACTCCCTGGAACTTCGAGAAAGTCGTGGATAAGGG
GGCCTCTGCCCAGTCCTTCATCGAAAGGATGACTAACTTTGATAAAAATCTGCCTAACGAAAAGGTGC
TTCCTAAACACTCTCTGCTGTACGAGTACTTCACAGTTTATAACGAGCTCACCAAGGTCAAATACGTCA
CAGAAGGGATGAGAAAGCCAGCATTCCTGTCTGGAGATCAGAAGAAAGCTATCGTGGACCTCCTCTT
CAAGACGAACCGGAAAGTTACCGTGAAACAGCTCAAAGAAGACTATTTCAAAAAGATTGAATGTTTC
GACTCTGTTGAAATCAGCGGAGTGGAGGATCGCTTCAACGCATCCCTGGGAACGTATCACGATCTCC
TGAAAATCATTAAAGACAAGGACTTCCTGGACAATGAGGAGAACGAGGACATTCTTGAGGACATTGT
CCTCACCCTTACGTTGTTTGAAGATAGGGAGATGATTGAAGAACGCTTGAAAACTTACGCTCATCTCT
TCGACGACAAAGTCATGAAACAGCTCAAGAGGCGCCGATATACAGGATGGGGGCGGCTGTCAAGAA
AACTGATCAATGGGATCCGAGACAAGCAGAGTGGAAAGACAATCCTGGATTTTCTTAAGTCCGATGG
ATTTGCCAACCGGAACTTCATT

Supplementary References

1. Ewen-Campen, B. *et al.* Optimized strategy for in vivo Cas9-activation in *Drosophila*. *Proc. Natl. Acad. Sci.* 201707635 (2017). doi:10.1073/pnas.1707635114

2. Waters, A. J. *et al.* Rationally-engineered reproductive barriers using CRISPR & CRISPRa: an evaluation of the synthetic species concept in Drosophila melanogaster. *Sci. Transl. Med.* (2018). doi:10.1101/259010

3. Lin, S., Ewen-Campen, B., Ni, X., Housden, B. E. & Perrimon, N. In vivo transcriptional activation using CRISPR/Cas9 in Drosophila. *Genetics* **201**, 433–442 (2015).

4. Lindsley, D. L., Grell, E. H. & Bridges, C. B. (Calvin B. *Genetic variations of Drosophila melanogaster*. (1967).

5. Ryder, E., Hartl, D. L. & Wu, C. -tin. The DrosDel Collection: A Set of P-Element Insertions for Generating Custom Chromosomal Aberrations in Drosophila melanogaster. *Genetics* **167**, 797–813 (2004).

6. Venken, K. J. T., He, Y., Hoskins, R. A. & Bellen, H. J. P[acman]: a BAC transgenic platform for targeted insertion of large DNA fragments in D. melanogaster. *Science* **314**, 1747–51 (2006).

7. Gratz, S. J. *et al.* Genome engineering of Drosophila with the CRISPR RNA-guided Cas9 nuclease. *Genetics* **194**, 1029–1035 (2013).

8. Port, F., Chen, H. M., Lee, T. & Bullock, S. L. Optimized CRISPR/Cas tools for efficient germline and somatic genome engineering in Drosophila. *Proc. Natl. Acad. Sci. U. S. A.* **111**, E2967-76 (2014).

9. Chavez, A. *et al.* Comparison of Cas9 activators in multiple species. *Nat. Methods* **13**, 563–7 (2016).

10. Stern, D. Addgene: p{CFD4-3xP3::DsRed}. Available at: https://www.addgene.org/86864/. (Accessed: 26th February 2020)
