# Supplementary material for: Engineering multiple species-like genetic incompatibilities in insects
Source: Nat Commun. 2020 Sep 8;11:4468. doi: 10.1038/s41467-020-18348-1 (PMC7478965; doi:10.1038/s41467-020-18348-1)
Supplement: Supplementary file 2 — Description of Additional Supplementary Files [file 41467_2020_18348_MOESM2_ESM.pdf]

Description of Additional Supplementary Files

File Name: Supplementary Movie 1

Description: An embedded time-lapse video of representative mating from **Fig. 3**. EGI flies are *pyr.Pfoxo*.Injection genotype. Images were taken on a Canon EOS Rebel T5 Digital SLR equipped with a Satechi remote shutter. Image files were compiled into movie (with shake correction) using Adobe Premier Pro 2019. For the purpose of the video, lights were left on for all 24 hours, 15 days of the experiment. During experiments reported in the paper, normal light-dark cycles were used, as described above.
