# Supplementary material for: Engineering multiple species-like genetic incompatibilities in insects
Source: Nat Commun. 2020 Sep 8;11:4468. doi: 10.1038/s41467-020-18348-1 (PMC7478965; doi:10.1038/s41467-020-18348-1)
Supplement: Supplementary file 4 — Reporting Summary [file 41467_2020_18348_MOESM4_ESM.pdf]

nature research

Corresponding author(s): Michael Smanski
Last updated by author(s): 2020-8-12

Reporting Summary

Nature Research wishes to improve the reproducibility of the work that we publish. This form provides structure for consistency and transparency in reporting. For further information on Nature Research policies, see our Editorial Policies and the Editorial Policy Checklist.

Statistics

For all statistical analyses, confirm that the following items are present in the figure legend, table legend, main text, or Methods section.

n/a	Confirmed	
☐	☒	The exact sample size (*n*) for each experimental group/condition, given as a discrete number and unit of measurement
☒	☐	A statement on whether measurements were taken from distinct samples or whether the same sample was measured repeatedly
☒	☐	The statistical test(s) used AND whether they are one- or two-sided<br>*Only common tests should be described solely by name; describe more complex techniques in the Methods section.*
☒	☐	A description of all covariates tested
☒	☐	A description of any assumptions or corrections, such as tests of normality and adjustment for multiple comparisons
☒	☐	A full description of the statistical parameters including central tendency (e.g. means) or other basic estimates (e.g. regression coefficient) AND variation (e.g. standard deviation) or associated estimates of uncertainty (e.g. confidence intervals)
☒	☐	For null hypothesis testing, the test statistic (e.g. *F*, *t*, *r*) with confidence intervals, effect sizes, degrees of freedom and *P* value noted<br>*Give P values as exact values whenever suitable.*
☒	☐	For Bayesian analysis, information on the choice of priors and Markov chain Monte Carlo settings
☒	☐	For hierarchical and complex designs, identification of the appropriate level for tests and full reporting of outcomes
☒	☐	Estimates of effect sizes (e.g. Cohen's *d*, Pearson's *r*), indicating how they were calculated

Our web collection on statistics for biologists contains articles on many of the points above.

Software and code

Policy information about availability of computer code

Data collection	The following software was used during data collection, processing, and communication associated with this study: ImageJ, Adobe Premier Pro 2019, Adobe Illustrator 2019, Python 3.2 (with Anaconda standard packages), Microsoft Excel and Word, Adobe Acrobat Pro
Data analysis	ImageJ and Python 3.2 were used in data analysis.

For manuscripts utilizing custom algorithms or software that are central to the research but not yet described in published literature, software must be made available to editors and reviewers. We strongly encourage code deposition in a community repository (e.g. GitHub). See the Nature Research guidelines for submitting code & software for further information.

Data

Policy information about availability of data

All manuscripts must include a data availability statement. This statement should provide the following information, where applicable:

- Accession codes, unique identifiers, or web links for publicly available datasets
- A list of figures that have associated raw data
- A description of any restrictions on data availability

All raw data is included in the Source Data File. Accession codes to key plasmid resources are pending and will be provided in the manuscript.

Field-specific reporting

Life sciences study design

All studies must disclose on these points even when the disclosure is negative.

Sample size	Sample sizes for each replicate of fly mating experiments were determined by population sizes supported in conventional Drosophila husbandry containers, as described in the book "Drosophila: a laboratory handbook" (Ashburner 1989)
Data exclusions	There were no data exclusions. On the contrary, even negative data (i.e. data collected for fly lines later determined to have a low frequency of balancer chromosomes) is retained and explained in the results.
Replication	We performed n=3 independent replicates of mating experiments, and replicated the entire process of strain engineering to create incompatible lines 11 times.
Randomization	Experimental randomization was not relevant to this study, as matings were required between specific combinations of genotypes to assess the performance of our approach. However, flies were chosen randomly from bottles of nominally isogenic flies to participate in mating trials.
Blinding	Blinding was not peformed, because experimental effects were not objective (alive vs dead) so subjective bias was not a worry.

Reporting for specific materials, systems and methods

We require information from authors about some types of materials, experimental systems and methods used in many studies. Here, indicate whether each material, system or method listed is relevant to your study. If you are not sure if a list item applies to your research, read the appropriate section before selecting a response.

Materials & experimental systems

n/a	Involved in the study	
☐	☒	Antibodies
☒	☐	Eukaryotic cell lines
☒	☐	Palaeontology and archaeology
☐	☒	Animals and other organisms
☒	☐	Human research participants
☒	☐	Clinical data
☒	☐	Dual use research of concern

Methods

n/a	Involved in the study	
☒	☐	ChIP-seq
☒	☐	Flow cytometry
☒	☐	MRI-based neuroimaging

Antibodies

Antibodies used	Drosophila-Patched, apal (Developmental Studies Hybridoma Bank (DSHB)) (1:50); Drosophila-Wingless, 4D4 (DSHB) (1:50); Drosophila-Armadillo, N2-7Al (DSHB) (1:50); Phospho-MAPK (ERKl/2), #4370 (Cell Signaling Technologies) (1:100). AlexaFluor 568 and 647 (Invitrogen) conjugated secondary antibodies were used as necessary at (1:500) dilution.
Validation	Validation data is available on the respective datasheets found in the following links: https://dshb.biology.uiowa.edu/Drosophila-Ptc-Apa-1; https://dshb.biology.uiowa.edu/4D4; https://dshb.biology.uiowa.edu/N2-7A1-Armadillo; https://www.cellsignal.com/products/primary-antibodies/phospho-p44-42-mapk-erk1-2-thr202-tyr204-d13-14-4e-xp-rabbit-mab/4370

Animals and other organisms

Policy information about studies involving animals; ARRIVE guidelines recommended for reporting animal research

Laboratory animals	Drosophila melanogaster w1118 was used as the parent to genetically engineered strains. w1118 and Oregon R were used for mating studies. Both Male and Female animals were used. Newly eclosed flies (with in 2 hours of eclosion) were selected for mating studies.
Wild animals	No wild animals were used in this study
Field-collected samples	No field-collected samples were used in this study
Ethics oversight	All work in this paper was approved by the University of Minnesota Institutional Biosafety Committee.

Note that full information on the approval of the study protocol must also be provided in the manuscript.
